# Metastable phase formation of Pt-X (X = Ir, Au) thin films

**DOI:** 10.1038/s41598-018-28452-4

**Published:** 2018-07-05

**Authors:** Aparna Saksena, Yu-Chuan Chien, Keke Chang, Pauline Kümmerl, Marcus Hans, Bernhard Völker, Jochen M. Schneider

**Affiliations:** 1 Materials Chemistry, RWTH Aachen University, 52074 Aachen, Germany; 2 Engineering Laboratory of Nuclear Energy Materials, Ningbo Institute of Materials Technology and Engineering, Chinese Academy of Sciences, 315201 Ningbo, Zhejiang China; 3 Max Planck Institut für Eisenforschung GmbH, Max-Planck-Strasse 1, 40237 Düsseldorf, Germany

## Abstract

The dependence of phase formation and mechanical properties on the chemical composition has been investigated for Pt-Ir and Pt-Au combinatorial thin films. The formation of a single, metastable Pt-Ir solid solution has been observed for all experimental compositions and temperatures. Upon Ir addition to Pt the experimentally determined changes in lattice parameter and Young’s modulus display rule of mixture behavior which is in good agreement with our *ab initio* data. Whereas, in the Pt-Au system, the single metastable solid solution decomposes into two phases as the growth temperature is raised to ≥600 °C. The lattice parameters in the dual phase region are independent of chemical composition. The substrate temperature and chemical composition dependent phase formation in Pt-Ir and Pt-Au thin films can be rationalized based on CALPHAD (CALculation of PHAse Diagrams) results combined with estimations of the activation energy required for surface diffusion: The metastable phase formation during film growth is caused by kinetic limitations, where Ir atoms (in Pt-Ir) need to overcome an up to factor 6 higher activation energy barrier than Au (in Pt-Au) to enable surface diffusion.

## Introduction

Pt-Ir alloys exhibit superior corrosion resistance^1–3^ in combination with attractive mechanical properties^1,3,4^. While Pt is corrosion resistant in most oxidizing environments, alloying with Ir drastically improves the mechanical properties rendering the alloy suitable for applications which demand high strength at elevated temperatures in corroding environments^1,4^. These alloys are thus employed in chemically demanding applications for example as electrode materials in fuel cells^5,6^, as catalyst in hydrogenation applications^7^ and organic chemical synthesis^8,9^, as protective coating for precision glass molding (PGM) applications^10–13^, as well as implantable electronic devices^14^, as a coronary stent^15,16^ and for many other medical applications^17^.

In 1930, Pt-Ir was reported to be miscible over the whole composition range by Darling^1^ based on the assessment of Nemilow and Fuessner *et al*.^18,19^. Electrical resistivity measurements by Zvyagintsev *et al*.^1,20^ also indicated the formation of a homogeneous solid solution during powder metallurgical process. In 1959, Raub and Plate^21^ proposed the presence of a miscibility gap at approximately 977 °C in Pt-Ir based on metallographic investigations, and presented electrical resistivity measurements, X-ray diffraction and dilatometric measurements in support of this notion. The miscibility gap reported thereafter was based on thermodynamic calculations^22,23^ and was inconsistent in shape with the report of Raub and Plate^21^. However, in 2009 Yamabe-Mitarai *et al*.^24^ experimentally refuted the existence of a miscibility gap in Pt-Ir at 1027 °C. The authors stated that for homogenized Pt_x_Ir_1−x_ (x = 0.25, 0.5, 0.65, 0.75) alloys, 1000 °C is too low to obtain phase segregation when annealing for 1000 hours in vacuum. Today, the controversy regarding phase formation in Pt-Ir appears to be unresolved.

For application in precision glass molding, the molding tool is required to be chemically stable under high mechanical loads during glass contact at temperatures of typically 400–700 °C^25,26^. Hence, despite of the high cost, the benchmark tool coating system utilized to meet these requirements is Pt_3_Ir_7_. To facilitate adhesion between the Pt_3_Ir_7_ protective coating and tungsten carbide tools, a metallic interlayer of for example chromium or nickel^27^ is commonly employed. Due to the harsh operating conditions during the glass molding process, the lifetime of the tool is limited^28,29^. Peng *et al*. have recently reported the temporal degradation behavior of the bi-layered coating (Cr or Ni adhesion layer + Pt_3_Ir_7_). Oxygen partial pressure dependent diffusion of the adhesion layer species^30^ along the Pt-Ir grain boundaries to the coating surface was identified as primary degradation mechanism. However, the ambiguity regarding the phase stability of Pt-Ir solid solutions may also be relevant in the context as thermally induced decomposition is expected to affect the grain boundary density even in non-reactive atmosphere.

However, the previously discussed ambiguity regarding the phase stability of Pt-Ir solid solutions may also be relevant in the context of precision glass molding motivating the here reported combinatorial phase formation investigation utilizing in addition to standard structure and composition analysis also atom probe tomography to determine the local chemical composition and potential variations thereof at the nm scale. Combinatorial depositions in combination with modern electronic structure predictions have previously been demonstrated to be an efficient method for rapid screening for binary alloys^31,32^ as well as for high entropy alloys^33^ regarding phase formation and phase stability.

Hence, we seek to identify the key factor defining the phase formation in Pt-X (X = Ir, Au) thin films. The experimentally determined phase formation in Pt-Ir and Pt-Au thin films is compared to phase formation predictions based on CALPHAD and *ab initio* calculations. Pt-Au is chosen as a reference system since the formation of a miscibility gap at 1300 °C in this system is not disputed in the literature^34,35^.

## Results and Discussion

**Figure 1:**
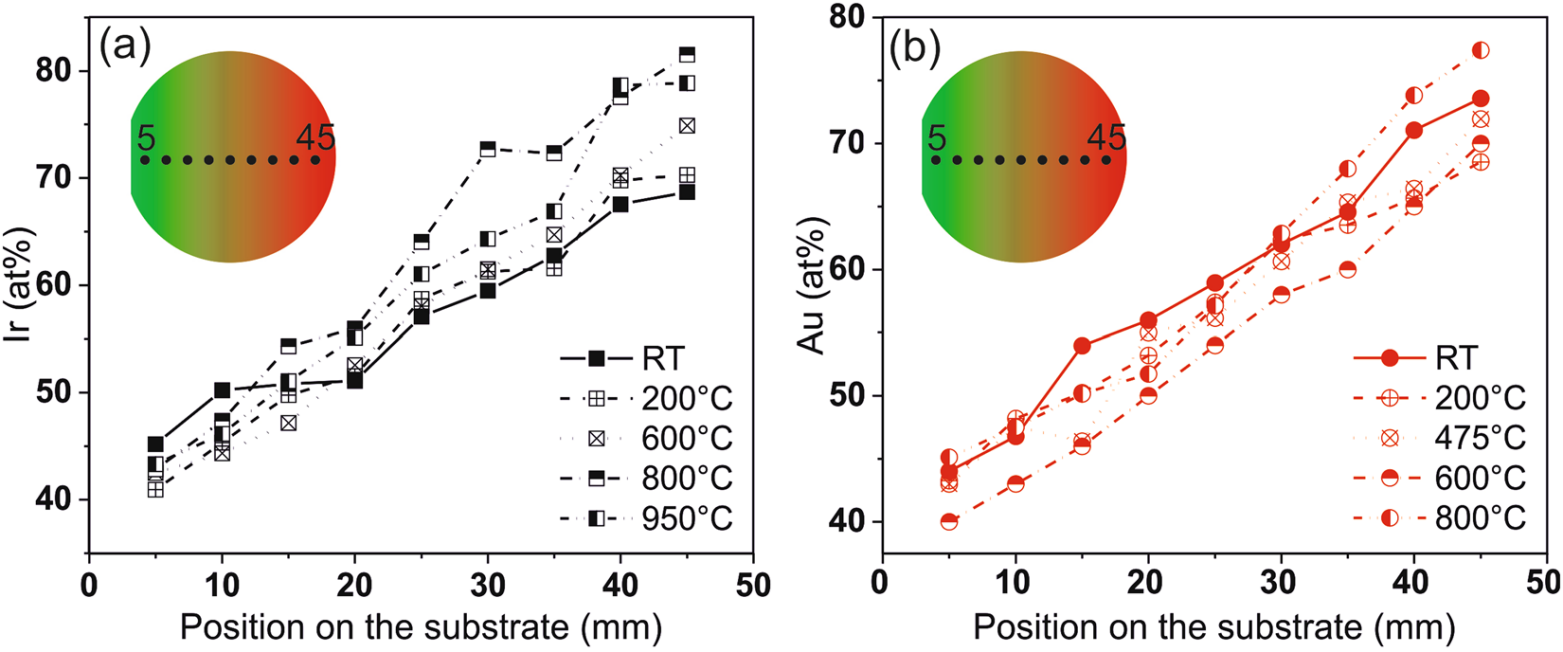
Chemical composition of the as grown Pt-Ir (**a**) and Pt-Au (**b**) thin films as a function of position on the substrate for different substrate temperature.

Figure 1(a,b) show the chemical composition of Pt-Ir and Pt-Au for different deposition temperatures, as a function of position on the substrate. It can be seen that the composition gradients range from approximately 40 to 80 at.% for all thin films.

**Figure 2:**
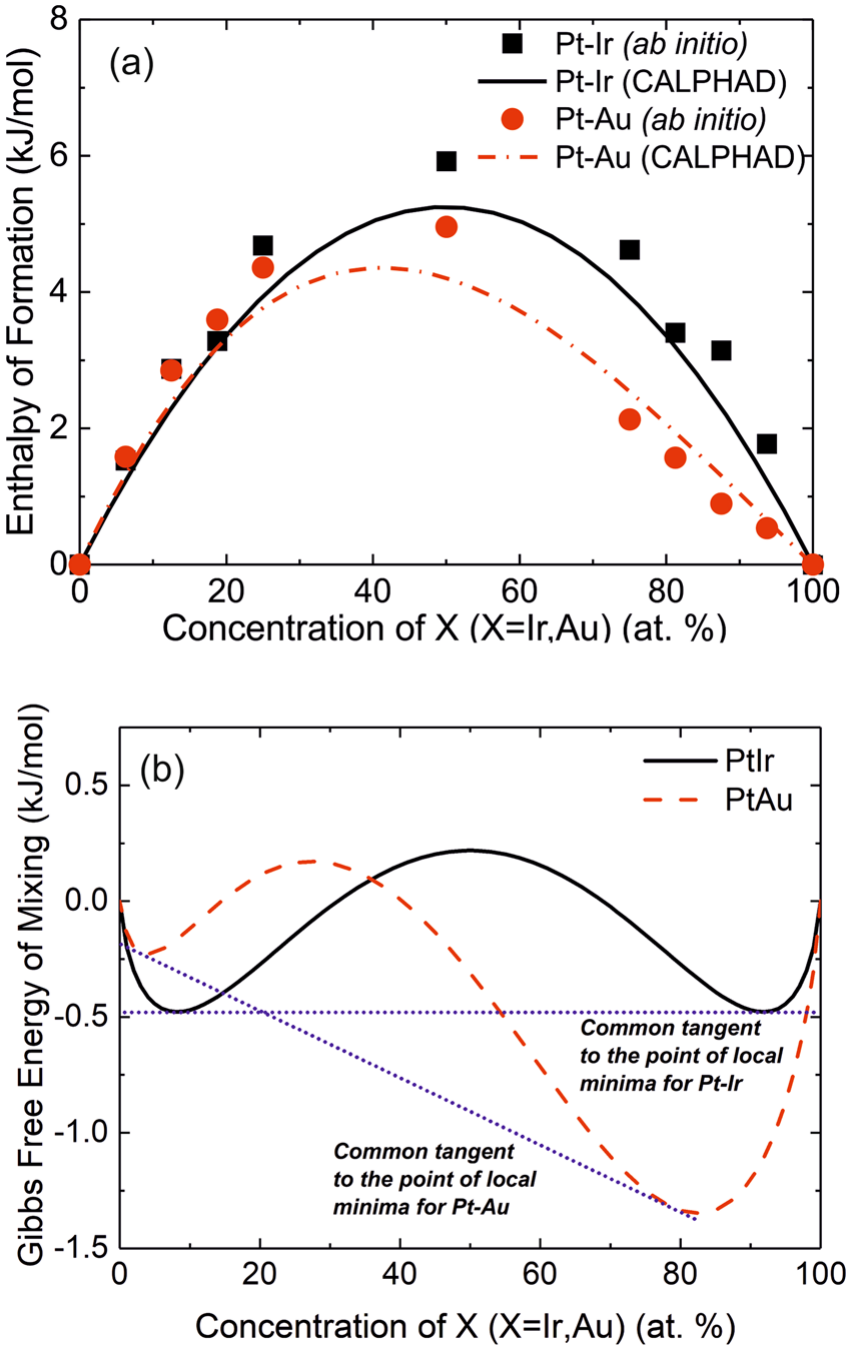
(**a**) Enthalpy of formation of Pt-Ir and Pt-Au systems calculated by ab initio and CALPHAD methods as a function of chemical composition, (**b**) CALPHAD determined Gibbs free energy of mixing data as a function of chemical composition at a temperature of 600 °C.

Calculations were performed for both Pt-X systems to predict the phase formation as a function of composition. The enthalpy of formation of Pt-X (Ir/Au) obtained by *ab initio* calculations at −273 °C and the standard enthalpy of formation calculated by CALPHAD at 25 °C are presented for comparison. Reasonable agreement is obtained, see Fig. 2(a), indicating endothermic mixing enthalpies over the complete composition range. Taking 600 °C as the basis of further calculations, CALPHAD calculated Gibbs free energy of mixing data is shown in Fig. 2(b). Both, the Pt-Ir and Pt-Au systems clearly show miscibility gap in the common tangent construction. Hence, these calculations suggest similar thermodynamic behavior for Pt-Ir and Pt-Au. Therefore, no significant difference in phase formation is expected. It is evident that the composition range of the as deposited thin films (Fig. 2(a,b)) lies within the miscibility gap.

**Figure 3:**
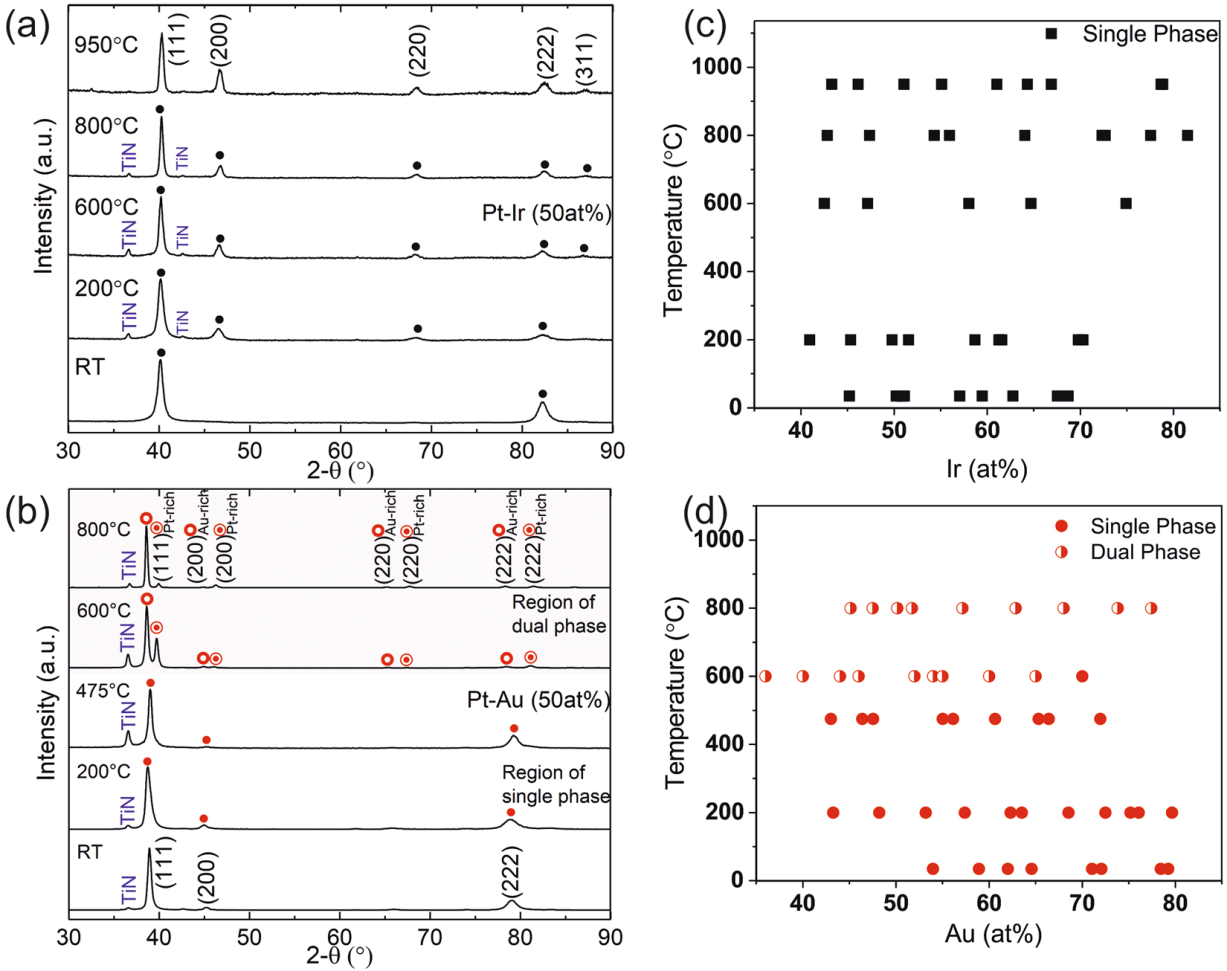
X-ray diffraction patterns of the as deposited Pt_50_Ir_50_ (**a**) and Pt_50_Au_50_ (**b**) for different deposition temperatures. All XRD data are compiled as a function of composition and the substrate temperature in the phase formation diagram for Pt-Ir (**c**) and Pt-Au (**d**) films.

Structural analysis was carried out for the thin film composition spreads that were deposited at different substrate temperatures. Composition regions of single and dual phases were identified from the X-ray diffraction patterns. Figure 3(a,b) show the diffraction patterns v/s growth temperature for a constant Pt_50_X_50_ (X = Ir, Au) composition. All XRD data are compiled in the phase formation diagram shown in Fig. 3(c,d) for Pt-X (X = Ir, Au) respectively. While Pt-Ir forms a solid solution independent of the growth temperature, decomposition of the solid solution phase in the Pt-Au system is observed at substrate temperatures ≥ 600 °C. The experimentally determined phase formation for Pt-Au is in agreement with the CALPHAD calculations presented in Fig. 2(b). However, for Pt-Ir it is evident that the experimental phase formation data (Fig. 3(c)) is clearly not in agreement with the thermodynamical phase formation prediction (Fig. 2(b)).

**Figure 4:**
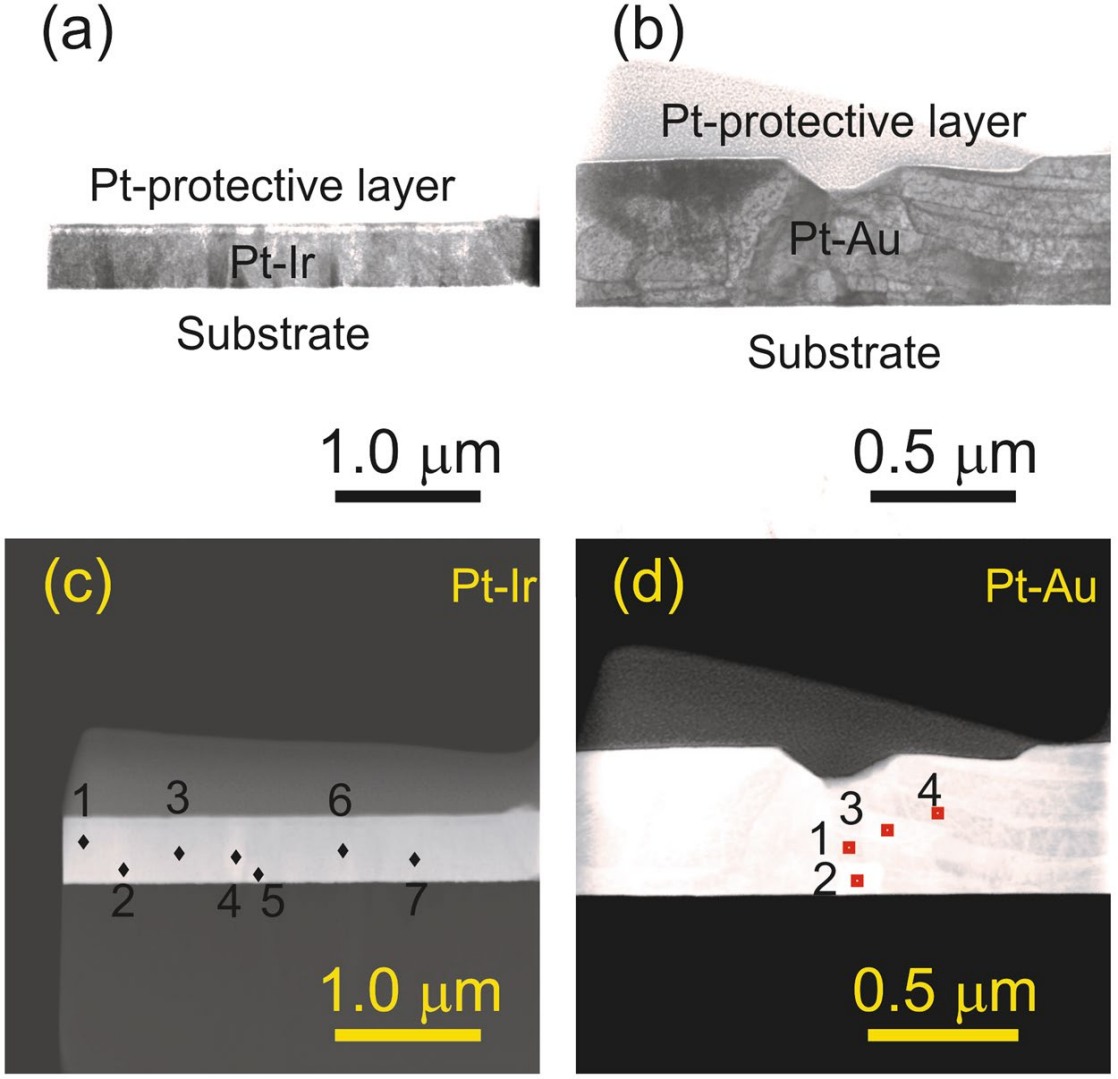
STEM images of Pt_42_Ir_58_ (**a**) and Pt_48_Au_52_ (**b**) taken as the representative sample for the Pt-X system (X = Ir, Au), deposited at 600 °C. Pt_42_Ir_58_ (**c**) and Pt_48_Au_52_ (**d**) is imaged in HAADF mode to visualize the contrast difference originated from compositional fluctuations.

The thin film morphology was characterized by cross sectional STEM analysis. Figure 4(a,b) show lamellae extracted from Pt_42_Ir_58_ and Pt_48_Au_52_ thin films deposited at 600 °C. The Pt_42_Ir_58_ thin film displays columnar grain growth whereas the Pt_48_Au_52_ morphology is characterized by faceted grain growth.

Figure 4(c,d) show the cross section of Pt_42_Ir_58_ and Pt_48_Au_52_ imaged in High Angle Annular Dark Field (HAADF) mode where contrast differences originate from difference in chemical composition within the probed region. No significant contrast variations could be observed in Pt_42_Ir_58_ indicating a homogeneous chemical composition. Chemical composition analysis by EDX within a probed region of approximately 10 nm radius on a 80 nm thick lamella, is performed at different locations within the cross section, as marked in Fig. 4(c). The variations in Pt concentration is within 42 at.% (Table 1), considering the measurement error of 1.1 at.%. This is in good agreement with the experimental observations.

**Table 1:** Composition of the regions marked in Fig. 4(c,d) for Pt_42_Ir_58_ and Pt_48_Au_52_ STEM lamellae.

Regions	Pt-Ir thin film (Ir = 58 at.%)		Pt-Au thin film (Au = 52 at.%)	
	Pt (at.%)	Ir (at.%)	Pt (at.%)	Au (at.%)
1	43	57	27	73
2	43	57	83	17
3	41	59	95	3
4	41	59	29	71
5	41	59		
6	43	57		
7	43	57		

For Pt_48_Au_52_ significant contrast fluctuation where lighter and darker regions correspond to individual grains can be seen. The darker region is the Au-rich phase while the lighter region represents the Pt-rich phase as confirmed by the compositional analysis in Table 1. It should be noted that the maximum composition gradient observed due to the combinatorial setup, over a 40 mm substrate is 32.72 at.% which corresponds to approximately 0.82 × 10^–3^ at.% µm^−1^. The dimension of the lamella for Pt-Ir is 460 nm × 80 nm × 3.32 µm. Therefore, the maximum composition variation over 3.32 µm is 0.0027 at.% and hence significantly smaller than the measurement error.

**Figure 5:**
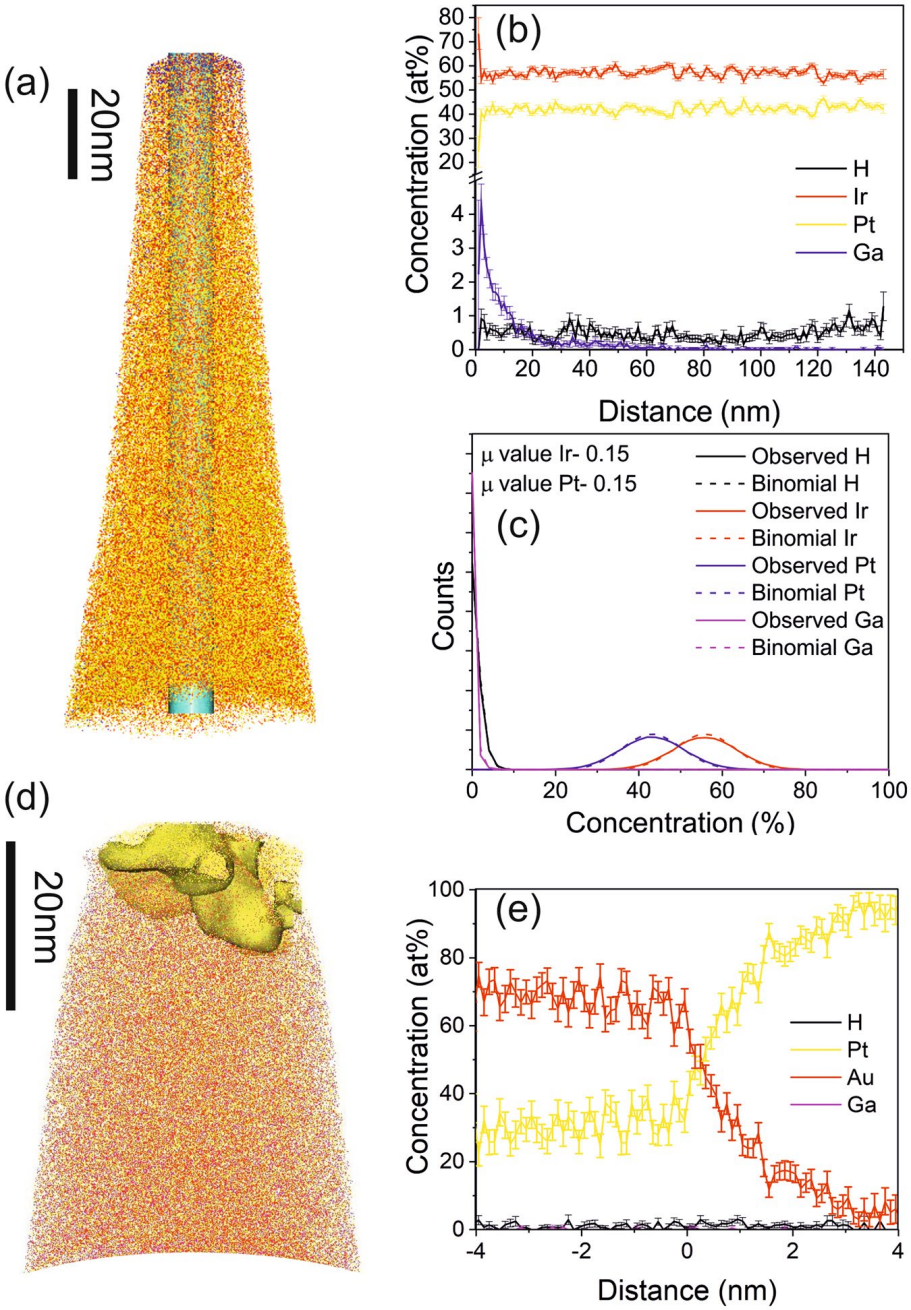
(**a**) Elemental distribution of the Pt_45_Ir_55_ thin film as deposited at 600 °C substrate temperature, (**b**) one dimensional concentration profile and a corresponding binomial distribution is represented in (**c**) indicating a random distribution of the solid solution. (**d**) Elemental distribution of the Pt_45_Au_55_ thin film, deposited at 600 °C substrate temperature where a proxigram at the interface between the two phases is represented in (**e**).

Analysis of the local chemical composition of Pt_45_Ir_55_ and Pt_50_Au_50_ thin films, deposited at 600 °C, was carried out by atom probe tomography (APT). Figure 5(a) shows the elemental distribution and Fig. 5(b) the one dimensional concentration profile of the Pt_45_Ir_55_ thin film in growth direction. Consistent with the HAADF investigations (Fig. 4(c)) the APT data compiled in Fig. 5(a,b), indicates a homogenous distribution of Pt and Ir. This notion is corroborated by the measured frequency distribution of the elements which is compared to the binomial distribution of randomly distributed elements shown in Fig. 5(c). The Pearson correlation coefficient µ is calculated and the value is found to be close to 0, indicating a close to random distribution of the elements. This is consistent with the formation of a single solid solution phase in line with the X-ray diffraction data (Fig. 3(a)).

The elemental distribution measured for the Pt_50_Au_50_ thin film is shown in Fig. 5(d) where the formation of two phases with different composition can be observed. In Fig. 5(e) the proxigram across the interface is shown indicating the formation of an Au-rich phase and Pt-rich phase. In contrast to the Pt-Ir thin film the nm scale observations for Pt-Au are consistent with previously discussed X-ray diffraction data (Fig. 3(b)) as well as the theoretical phase formation predictions based on CALPHAD (Fig. 2).

**Figure 6:**
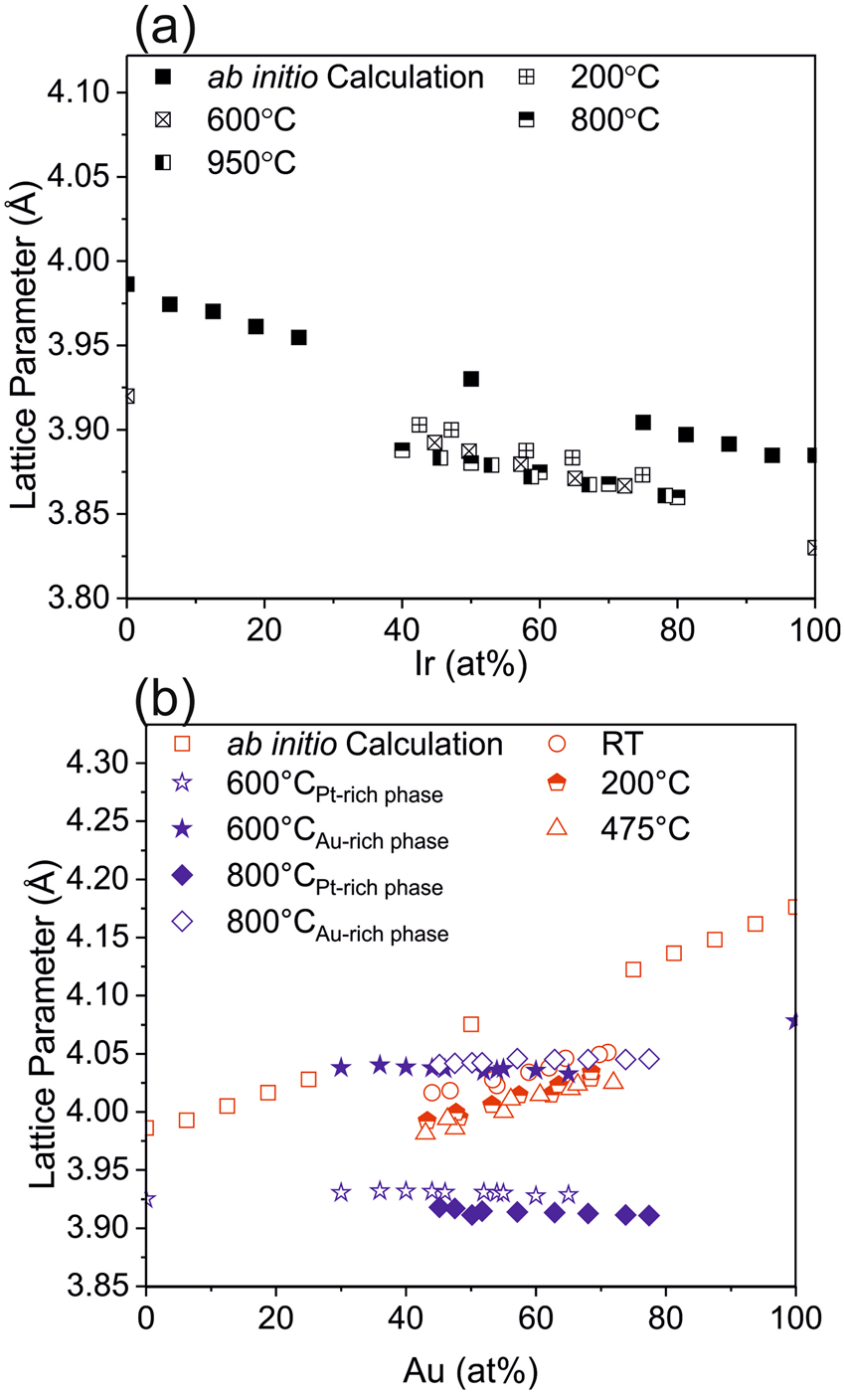
(**a**) Lattice parameters of the Pt-Ir thin film showing no phase separation for the different deposition temperatures is compared with the lattice parameter calculated by ab initio calculations, (**b**) Lattice parameters of the single phase Pt-Au thin films at 475 °C and below, and phase separated Pt-Au thin films for 600 °C and above, as deposited films, compared with the lattice parameters calculated via ab initio calculations.

To critically appraise the *ab initio* predictions regarding lattice parameters and elastic moduli these data were compared to X-ray diffraction and nanoindentation data. Figure 6(a) shows the experimentally determined lattice parameters obtained for the Pt-Ir thin film composition spreads at different temperatures. While Pt and Ir both crystallize in an fcc structure (with the lattice parameters of 3.91Å^36^ and 3.84Å^37^, respectively), the fcc solid solution lattice expands as a consequence of addition of Pt which exhibits a 1.8% larger lattice parameter than Ir. The close to linear trend of experimentally determined lattice parameter as a function of composition is in a good agreement with the *ab initio* data, showing a deviation of ≤1.5%.

Figure 6(b) shows the experimentally determined lattice parameters for Pt-Au thin films, deposited at various temperatures. Similar to Pt and Ir, Au also crystallizes in an fcc structure with a lattice parameter of 4.07Å^36^. The experimental lattice parameters for all the films deposited at room temperature (without intentional heating), at 200 and at 475 °C increase linearly as a consequence of the lattice expansion upon incorporation of Au which exhibit a 4.1% larger lattice parameter than Pt. At these temperatures, the formation of the metastable single fcc phase is observed. The measured lattice parameters are in good agreement with *ab initio* predictions (deviation of ≤1.7%). At temperatures above 600 °C, the formation of two solid solution phases is observed. For both phases the lattice parameters are independent of the chemical composition.

**Figure 7:**
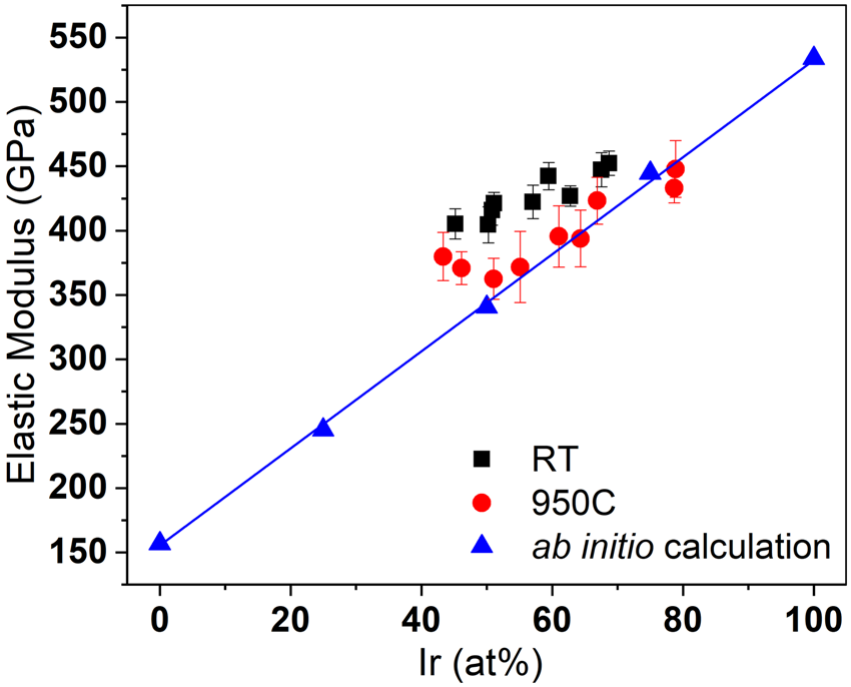
Elastic modulus of Pt-Ir thin films measured by nanoindentation for different deposition temperatures, compared with the ab initio calculations showing good agreement.

The elastic modulus of the Pt-Ir single phase is measured and compared with the theoretical calculations for the films deposited at different temperatures, as shown in the Fig. 7. The measured elastic moduli also agree well with the calculations performed (maximum deviation 18%). While the elastic modulus of Pt and Ir is 168 GPa and 528 GPa, the elastic modulus of the fcc solid solution increases linearly with the addition of Ir to the lattice, which constitutes a 214% higher elastic modulus.

While, no mechanical property measurements and calculations are performed for the Pt-Au system because of the formation of two phases, it is evident for Pt-Ir that the experimental lattice parameter and the elasticity data are consistent with the *ab initio* predictions. However, as pointed out above it is also evident that the experimental phase formation data for Pt-Ir (Fig. 3(c)) is clearly not in agreement with the thermodynamic phase formation prediction (Fig. 2(b)).

It may be speculated that this deviation from the thermodynamic predictions is due to kinetically limited thin film growth^38^. Depending on the substrate temperature, deposition rate and the activation energy required for surface diffusion^39^, adatom mobility^32^ may be limited resulting in the formation of a metastable phase^38^.

The formation of a metastable solid solution phase due to kinetically limited growth was observed in the Pt-Au system where at temperatures ≤475 °C the formation of a metastable solid solution phase was detected, while at substrate temperatures of 600 °C and above phase separation into the two predicted equilibrium phases could be perceived. However, in the Pt-Ir system kinetically limited growth was observed for all deposition temperatures. Hence, to understand the apparent disparity in phase formation between Pt-Au and Pt-Ir, the activation energy required for surface diffusion is calculated utilizing a previously published methodology^39^. A Pt atom is systematically moved on the (111) plane of Pt_x_Ir_1−x_ and of Pt_x_Au_1−x_ (where x = 0, 0.25, 0.5, 0.75, 1) in <110> direction to the neighboring site, because of the ease of movement in this direction for fcc systems. This process is repeated for an Ir and Au atom on the respective Pt_x_Ir_1−x_ and Pt_x_Au_1−x_ (where x = 0, 0.25, 0.5, 0.75, 1) (111) plane and the activation energy is calculated by the difference of maximum and minimum energies during this movement.

During film growth, the temperature dependent atomic mobility is governed by the following equation, proposed by Cantor and Cahn^40^.

1 $$X=\sqrt{2v\frac{a}{{r}_{D}}}\ast a\ast exp(-\frac{{Q}_{s}}{2kT})$$

where X is the diffusion distance, $v$ is the vibrational frequency of surface atoms (~10^13^ s^−1^), $a$ is the individual jump distance, ${r}_{D}$ is the deposition rate, ${Q}_{s}$ is the activation energy for surface diffusion, *k* is the Boltzmann constant and *T* is the substrate temperature during deposition.

**Figure 8:**
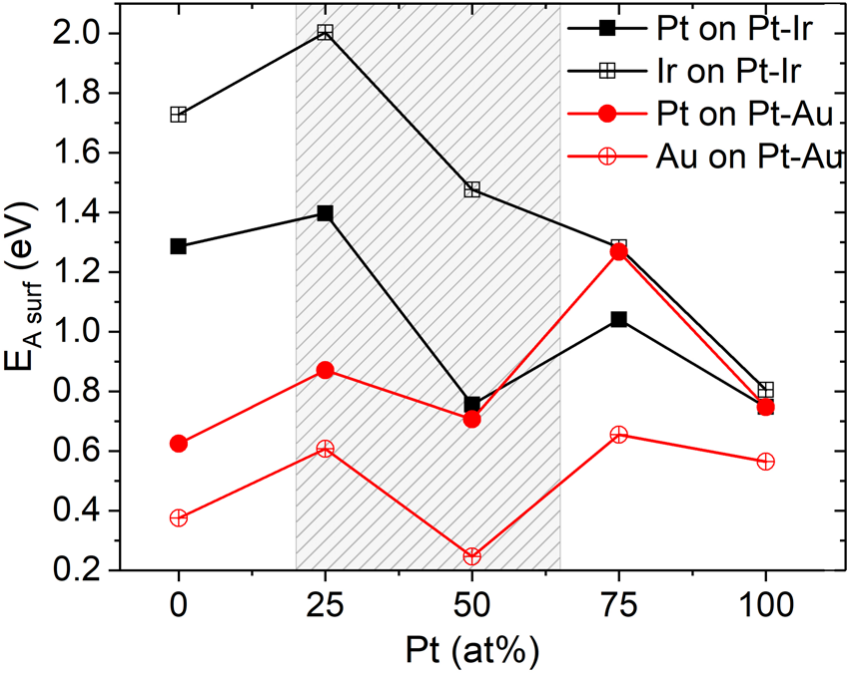
Activation energy for surface diffusion (E _A surf_) to reach the equilibrium state, obtained via ab initio calculations for an atom on respective Pt-Ir and Pt-Au (111) surface in <110> direction. The highlighted region depicts the experimentally investigated compositional range.

Hence, the diffusion distance X increases exponentially with temperature at a rate determined by Q_s_. Comparing surface migration of two different species at constant temperature the diffusion distance X decays exponentially with Q_s_. All calculated activation energies for surface diffusion Q_s_ are compiled in Fig. 8. Comparing Q_s_ for Pt on Pt_0.5_Ir_0.5_ with Q_s_ for Pt on Pt_0.5_Au_0.5_ the difference is 6% which is very minute. However, comparing Q_s_ for Ir on Pt_0.5_Ir_0.5_ with Q_s_ for Au on Pt_0.5_Au_0.5_ it is evident that a factor six times larger activation energy is required for the migration of Ir compared to Au.

Since the activation energy barrier for migration during film growth, is much higher in Pt-Ir than in Pt-Au, Pt-Ir forms a kinetically limited, metastable solid solution. For the Pt-Au system the kinetic limitation is overcome by temperature induced migration of Au on Pt_0.5_Au_0.5_ at deposition temperatures of ≥600 °C. Hence, based on these calculations the experimentally obtained phase formation data for the Pt-Ir and Pt-Au systems can be rationalized.

## Conclusions

Pt-Ir and Pt-Au thin films were deposited via combinatorial magnetron sputtering at various substrate temperatures. CALPHAD data predict a miscibility gap and hence the formation of two solid solution phases for Pt-Ir and Pt-Au systems. Consistent with this prediction experimental phase formation studies indicate the decomposition of the metastable Pt-Au solid solution phase at and above 600 °C. However, in contradiction to the phase formation prediction a metastable Pt-Ir solid solution is observed based on X-ray diffraction and supported by scanning transmission electron microscopy and atom probe tomography data within the substrate temperature range studied here. The experimental lattice parameter and the elasticity data are consistent with *ab initio* predictions for the metastable Pt-Ir solid solution. To rationalize the apparent disparity in phase formation between the two thin film material systems investigated here, the activation energy for migration during thin film growth was calculated and compared.

The activation energy required for surface diffusion of Ir on Pt-Ir is up to six times higher than the energy required for Au migration on Pt-Au. Hence, both, the decomposition of Pt-Au as well as the formation of the metastable Pt-Ir solid solution can be readily understood based on the relevant migration activation energy data.

## Methods

### Experimental methods

**Figure 9:**
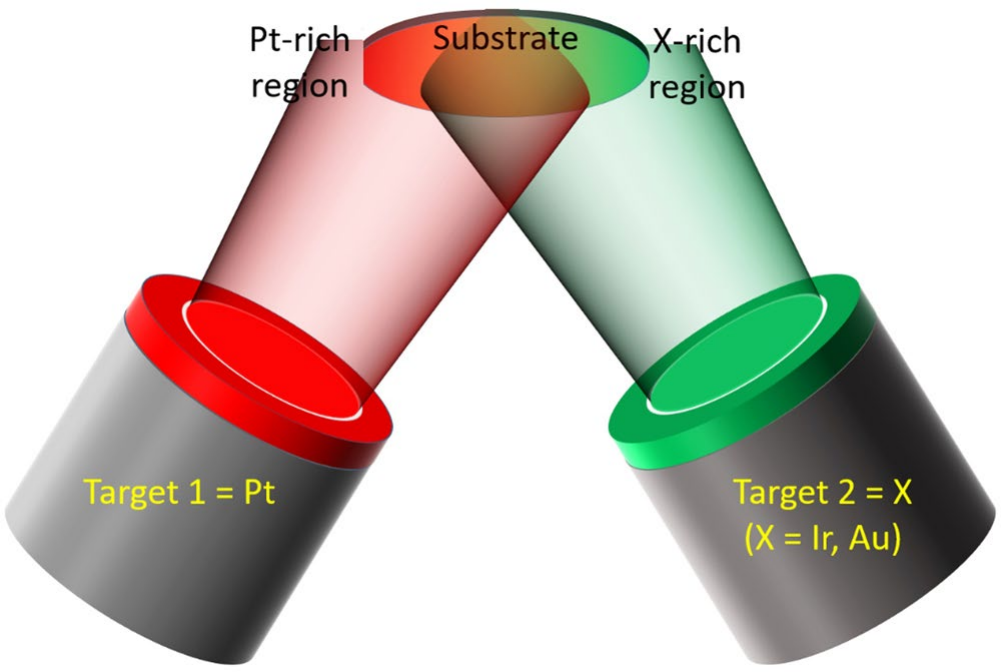
Schematics of the combinatorial deposition setup, the substrate illustrates the observed compositional gradient.

Pt-Ir and Pt-Au thin films were deposited using combinatorial magnetron sputtering. A schematic representation of the experimental setup is shown in the Fig. 9. The color gradient on the substrate indicates the Pt-X (X = Ir, Au) composition gradient. Thin films were synthesized at room temperature (without intentional heating) and at substrate temperatures of 200, 475, 600, 800 and 950 °C. All growth experiments were carried out in an argon atmosphere at a pressure of 0.4 Pa. The base pressure before the depositions was ≤7 × 10^−5^ Pa. Individual elemental targets of Pt (99.99% purity) and Ir (99.9% purity) or Au (99.99% purity) were used.

All the growth experiments were performed by high power impulse magnetron sputtering (HiPIMS) at a frequency of 500 Hz and a duty cycle of 2.5%. The average applied power densities were 4.5 W cm^−2^ on Ir and 2 W cm^−2^ on Pt for the growth of the Pt-Ir thin films while for the Pt-Au system a power density of 2.5 W cm^−2^ was employed for both magnetrons. The higher applied power density for Ir was required due to the 57% lower sputtering yield of Ir compared to Au, in order to deposit X-rich films (X = Ir, Au) with similar composition gradients. The substrate to target distance was maintained at 10 cm continuously for all the growth experiments. The ~600 nm thick Pt-X films were deposited onto 2 µm thick TiN layer coated sapphire (0001) substrates. TiN served as diffusion barrier and details of the film deposition were reported elsewhere^41^.

The compositional gradient of the as deposited films was analyzed by energy dispersive X-ray spectroscopy (EDX) using a JEOL JSM-6480 scanning electron microscope, equipped with an EDAX Genesis 2000 detector. X-ray emission was stimulated by 15 keV primary electron irradiation of the thin film samples at a working distance of 10 mm. The elemental composition was determined by evaluating the L spectra.

Spatially resolved compositional analysis was conducted by APT using a local electrode atom probe (LEAP 4000X HR™ Cameca Instrument) with laser assisted field evaporation to characterize the elemental composition information and the distribution thereof at nm scale. Measurements were performed at a specimen temperature of −213 °C with a laser energy of 40 pJ and a frequency of 125 kHz. The IVAS 3.6.10 software package was employed for data reconstruction and analysis.

Morphology and phase distribution were investigated utilizing a JEOL JSM-2200FS field emission gun in scanning transmission electron microscopy (STEM) mode at 200 kV acceleration voltage with a 30 mm^2^ area EDX silicon drift detector from JEOL. APT tips as well as STEM lamellae were extracted utilizing Ga ions in a FEI HELIOS Nanolab 660 focused ion beam (FIB) dual beam microscope.

Structure analysis of the as deposited thin films was performed using X-ray diffraction (XRD). A Bruker D8 general area detection diffraction system (GADDS) in grazing incidence geometry was used to perform 2θ scans at a grazing angle of 15°, using Cu Kα (λ = 1.54 Å) radiation. The composition of Pt-X thin film is measured as shown in Fig. 1, in a step size of 5 mm along the concentration gradient. Grazing angle XRD was carried out at these positions and the corresponding compositions were calculated based on the EDX data compiled in Fig. 1.

The elastic moduli of the thin films were obtained by using a Hysitron TriboIndenter TI-900 with an applied load of 1 mN on a Berkovich diamond indenter and the maximum contact depth was in the order of 50 nm. The reported values were an average of at least 9 indents per composition. The Poisson’s ratio ν was theoretically calculated by density functional theory (DFT) for the system Pt-X of the compositions, 0 at.%, 25 at.%, 50 at.%, 75 at.% and 100 at.% and these values were then interpolated to calculate the elastic modulus from the reduced modulus as a function of the experimentally measured compositions.

### Theoretical methods

First Principle calculations were performed with the Vienna *Ab Initio* Simulation Package (VASP). Generalized gradient approximation (GGA) was applied using projector augmented plane-wave potentials. The plane-wave cut off energy and the convergence criterion were set to be 500 eV and 10^−6^ eV respectively. The Blöchl corrections for the total energy were applied. Brillouin zone integrations were conducted on appropriate *k*-points determined by Monkhorst-Pack^42^. The energy at the equilibrium state was obtained from an additional static calculation. The structures were fully relaxed in terms of volume and atomic positions.

The lattice constant and bulk modulus of a pure element was determined by fitting the Birch-Murnaghan equation of state^43^. The energy of formation of binary systems were calculated with the 16-atom special quasi-random structures (SQS)^39^. The lattice constant was derived from the average volume per atom of the relaxed SQS-cell.

The activation energy for surface diffusion was calculated by moving an atom stepwise from its lattice site to the nearest neighboring site on the surface of the close packed plane (111) in <110> direction. The activation energy was determined by the difference of the maximum and minimum energies along this route. Static calculations of the steps were carried out in a 48-atom supercell whose energy per atom was closest to that of a SQS with identical composition.

Elastic constants, i.e., C11, C12 and C44, of the systems were conducted with the method published by Music *et al*.^44^ in 32-atom cubic supercells whose energy per atom was closest to that of a SQS with identical composition. The bulk modulus was calculated by fitting the Birch-Murnaghan equation of state with the energy-volume data points of the supercells. The Young’s modulus was derived from the elastic constants, where the details were presented by Reeh *et al*.^45^. The Poisson’s ratio was calculated from the bulk and Young’s moduli.

The thermodynamic assessment of the binary systems was carried out based on an extensive literature review. The CALPHAD approach was utilized to calculate stable phase diagrams employing the FactSage software^46^ and the thermodynamic datasets of Pt-Ir^47^ and Pt-Au^35^.

### Data availability

The data and samples analyzed during the current study are available from the corresponding author upon request.
